# Lack of evidence for ectopic sprouting of genetically labeled Aβ touch afferents in inflammatory and neuropathic trigeminal pain

**DOI:** 10.1186/s12990-015-0017-2

**Published:** 2015-04-10

**Authors:** Yi Zhang, Yong Chen, Wolfgang Liedtke, Fan Wang

**Affiliations:** Department of Neurobiology, Duke University Medical Center, Durham, NC 27710 USA; Department of Neurology, Center for Translational Neuroscience, Duke University Medical Center, Durham, NC 27710 USA

**Keywords:** Mechanical allodynia, Aβ fibers, Sprouting, Inflammatory pain, Neuropathic pain

## Abstract

**Background:**

Mechanical and in particular tactile allodynia is a hallmark of chronic pain in which innocuous touch becomes painful. Previous cholera toxin B (CTB)-based neural tracing experiments and electrophysiology studies had suggested that aberrant axon sprouting from touch sensory afferents into pain-processing laminae after injury is a possible anatomical substrate underlying mechanical allodynia. This hypothesis was later challenged by experiments using intra-axonal labeling of A-fiber neurons, as well as single-neuron labeling of electrophysiologically identified sensory neurons. However, no studies have used genetically labeled neurons to examine this issue, and most studies were performed on spinal but not trigeminal sensory neurons which are the relevant neurons for orofacial pain, where allodynia oftentimes plays a dominant clinical role.

**Findings:**

We recently discovered that parvalbumin::Cre (Pv::Cre) labels two types of Aβ touch neurons in trigeminal ganglion. Using a Pv::CreER driver and a Cre-dependent reporter mouse, we specifically labeled these Aβ trigeminal touch afferents by timed taxomifen injection prior to inflammation or infraorbital nerve injury (ION transection). We then examined the peripheral and central projections of labeled axons into the brainstem caudalis nucleus after injuries vs controls. We found no evidence for ectopic sprouting of Pv::CreER labeled trigeminal Aβ axons into the superficial trigeminal noci-receptive laminae. Furthermore, there was also no evidence for peripheral sprouting.

**Conclusions:**

CreER-based labeling prior to injury precluded the issue of phenotypic changes of neurons after injury. Our results suggest that touch allodynia in chronic orofacial pain is unlikely caused by ectopic sprouting of Aβ trigeminal afferents.

## Background

Mechanical allodynia, in which light touch is perceived as painful [1], is a hallmark of many chronic pain conditions. Numerous previous studies reported changes that were interpreted as ectopic collateral sprouting of Aβ touch afferents into the “pain-processing” superficial laminae I-II after nerve injury [2-6]. The abnormal sprouting was observed initially using bulk transport of cholera toxin B (CTB), and later using single-fiber labeling and electrophysiology [2,5,6]. The use of CTB as a specific marker taken up selectively by large-diameter Aβ neurons in the periphery was later found to be problematic [7-10]. Furthermore, intra-axonal recording followed by intra-axonal injection of the neural tracer into the identified Aβ fibers revealed a complete lack of evidence for sprouting of putative Aβ touch afferents (low-threshold mechanoreceptors, or LTMRs) into the superficial laminae [11]. Another evidence argue against Aβ fibers sprouting came from the finding that the plexus of VGLUT1-immunoreactive boutons in laminae IIi-III of the lumbar dorsal horn (most of which originate from Aβ LTMR afferents) did not extend more dorsally after sciatic nerve transection [12]. More recently, Koerber and colleagues used an ex vivo prep to record from the soma of previously axotomized peripheral neurons, then labeled the recorded neurons with intrasomal staining. This approach also demonstrated that individually labeled Aβ LTMR afferents do not sprout into “pain” laminae after injury, but instead, they discovered that myelinated nociceptive neurons (high threshold mechanoreceptors HTMRs) form flame-shaped terminal arbors that project throughout lamina I-V. They concluded that these myelinated HTMRs could account for the morphological findings previously assigned to sprouting LTMRs [13].

These previous studies were performed on dorsal root ganglion (DRG) sensory neurons. We want to examine trigeminal ganglion (TG) sensory neurons that innervate the head, face and its adjacent sentient structures such as teeth, sinuses, dura mater, cornea, temporo-mandibular joint (TMJ). TG sensory neurons are most relevant for orofacial pain and headaches, forms of pain that share a clinical hallmark of significant mechanical allodynia, e.g. in trigeminal neuralgia, atypical face pain, dental pulpitis, keratitis, TMJ disorder, postherpetic trigeminal nerve pain and migraine [14-21]. We therefore felt compelled to probe the neuro-anatomical basis of mechanical allodynia in the TG system. In animal models, TG nerve injury shares similarities with its DRG counterpart, but there are also significant differences [22]. Previously, trigeminal nerve transection studies had reported changes in central axon arborizations after transection, but these transection studies were performed in fetal or neonatal mice [23,24]. Similar experiments in adult animals had not been performed. Furthermore, we wanted to use a genetic method to label Aβ TG touch neurons of defined morphological types before injury, in order to determine for these precisely defined Aβ touch afferent whether they sprout ectopically into noci-receptive laminae in the brainstem after injury.

Painful orofacial cues are detected by nociceptive TG neurons and relayed to the brainstem trigeminal spinal nucleus, the spinal caudalis (SpC). Similarly to the spinal cord dorsal horn, C-fiber peptidergic and non-peptidergic TG nociceptive neurons project to the superficial layers of SpC including lamina I and lamina II [25], whereas TG LTMR neurons project to deeper laminae in SpC (III –V). Importantly, our recent experiments revealed that in the mouse TG system, parvalbumin::Cre (Pv::Cre) selectively labeled two types of Aβ touch (LTMR) neurons: primarily slowly adapting Merkel-ending neurons and a small number of rapidly adapting longitudinal lanceolate-ending neurons [26]. In the current study we also use Pv::Cre and Pv::CreER (in which tamoxifen-inducible CreER is knocked into the Pv locus) [26] mouse lines and subject them to two orofacial pain models in order to probe peripheral and central axonal projections of TG Aβ afferents in pathological conditions.

## Results and discussion

### Using neonatal tamoxifen injection in Pv-CreER; Ai14 mice to specifically label individual TG Aβ touch afferents

**Figure 1:**
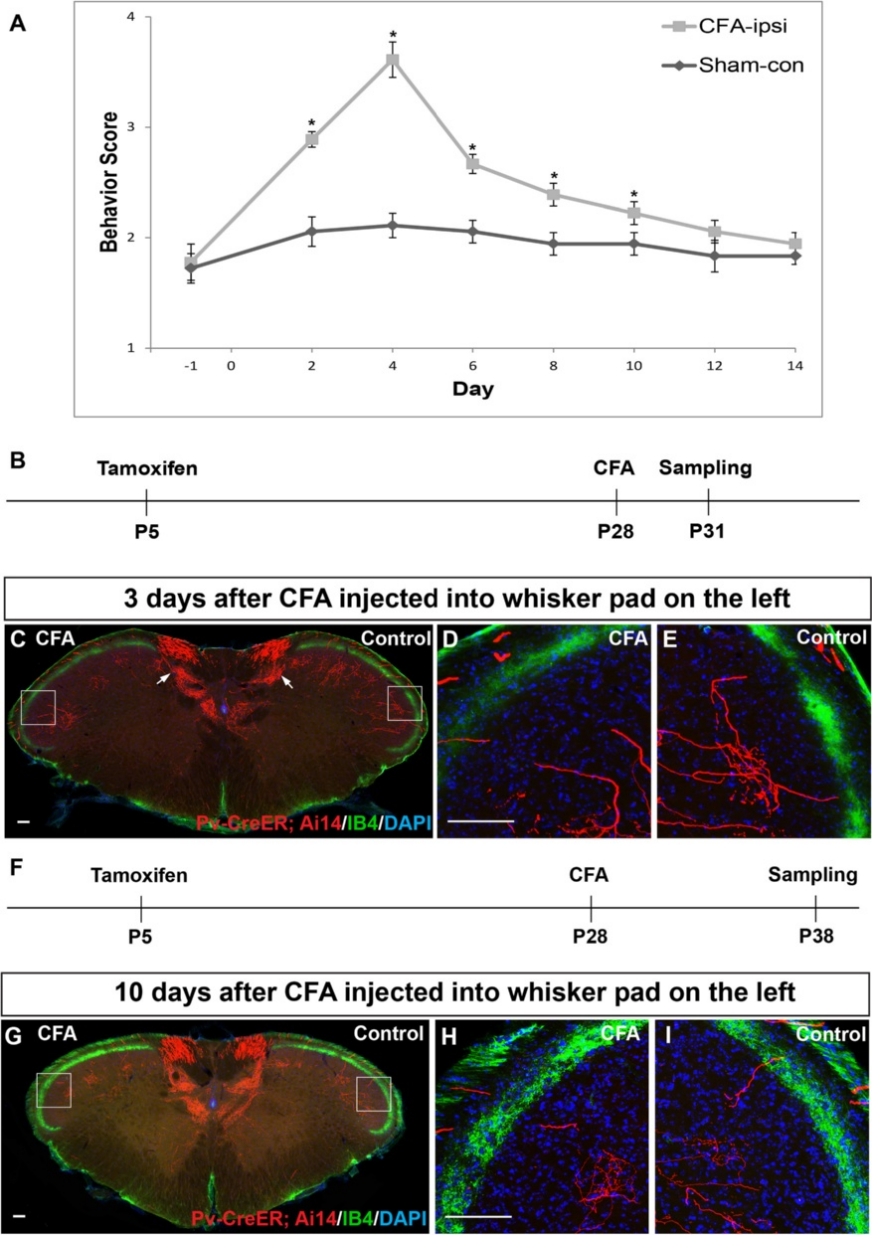
**CFA induced inflammation caused mechanical allodynia but not axon sprouting of labeled trigeminal Aβ afferents. (A)** Unilateral injection of CFA into whisker pad induced mechanical allodynia on the ipsilateral side. Higher behavior score indicated an increased aversive response to von Frey filament stimulation. Results represent means ± SEM. P-values represent comparison to sham values (*P < 0.05). Differences were determined by Student’s *t* test between two groups, or one-way ANOVA followed by post-hoc Bonferroni test for multiple groups. n = 6 mice for each group. **(B)** Schematic of experimental procedure for results shown in **C-E**. **(C)** Representative spinal caudalis (SpC) section from Pv-CreER; Ai14 transgenic mice. Red: labeled Aβ axon collaterals; Green: IB4-488 staining marked lamina II; Blue: DAPI. Left side is CFA injected side (3 days post injection), while right side is control IFA injected side. **(D-E)** High magnification images of boxed regions in **C**. **(F)** Schematic of experimental procedure for results shown in **G-I**. **(G)** Representative spinal caudalis (SpC) section from Pv-CreER; Ai14 transgenic mice. Red: labeled Aβ axon collaterals; Green: IB4-488 staining marked lamina II; Blue: DAPI. Left side is CFA injected side (10 days post injection), while right side is control IFA injected side. **(H-I)** High magnification of boxed regions in **G**. Scale bar: 100 μm.

We crossed Pv::CreER with the Ai14 reporter line in which the red fluorescent protein tomato is expressed in a Cre-dependent manner (Rosa-lox-STOP-lox-tomato) [27]. Although Pv is also expressed in second-order neurons in the brainstem trigeminal nuclei in addition to TG sensory neurons [28], this expression in brainstem begins only after postnatal day 7 (P7). Thus, in “Pv::CreER; Ai14”-mice, tamoxifen injected at P5 would only activate CreER in TG Aβ LTMR afferents, and selectively and permanently label these Aβ fibers with tomato, but not other neurons (Figure 1B-C). We then let these tamoxifen-injected mice develop to adult, and subjected them to two types of TG chronic pain models (described below), which allowed us to examine whether the labeled Aβ afferents ectopically sprout into the superficial layers in SpC. We noted that due to the moderate efficiency of CreER activation, only small numbers of TG Aβ fibers were labeled. This is not necessarily a disadvantage because the sparse labeling allowed high resolution imaging of individual terminal arbors (Figure 1C-E). Since Pv is also strongly expressed by proprioceptive DRG sensory neurons, CreER is also activated in these neurons by tamoxifen. As a result, tomato-labeled axonal collaterals from these proprioceptive neurons can be seen projecting into the dorsal column nuclei in the brainstem section (Figure 1C, arrows). These proprioceptive axons are not directly relevant for our studies here, so they will not be mentioned further.

### No evidence of ectopic central sprouting of TG Aβ touch afferents in a CFA-induced trigeminal inflammatory pain model

We first carried out experiments using an inflammatory pain model. Numerous previous studies showed that injection of complete Freund’s adjuvant (CFA) into the whisker pad (which are innervated by the infraorbital branch of the maxillary nerve) resulted in prolonged mechanical allodynia that lasted 10 days or longer [29-35]. We repeated the CFA inflammation experiments using the “Pv:CreER; Ai14”- mice after neonatal tamoxifen injection at P5. After CFA injection into the whisker pad, we used von Frey hairs to examine mechanical sensitivity at different time points. In keeping with previous experiences, we observed that 3 ~ 4 days following initial inflammatory injury, mechanical allodynia was most pronounced, and it lasted more than 10 days (Figure 1A). Thus, we chose to examine the axon projections at day 3 and day 10 post CFA injection, to determine whether there would be any transient ectopic sprouting (at day 3) or sprouting at late stage (at day 10) (Figure 1B and F, schematic of experimental procedure). CFA was injected into the left whisker pad of adult “Pv-CreER; Ai14”-mice (that had tamoxifen injection at P5). The whisker pad on the right side was injected with IFA and served as internal control. We used isolectin B4 (IB4) to mark the inner lamina of the “pain-processing” layers in SpC. IB4 is a marker for non-peptidergic nociceptive fibers that project to lamina II of SpC, same as for spinal sensory neurons [36]. Figure 1C-E shows representative images of SpC sections 3 days after CFA injection. Figure 1C is an overview of the SpC. The left side of the SpC is CFA-injected and the right side is control (Figure 1C). High magnification images from the regions receiving inputs from the whisker pad (boxed area) are shown in Figure 1D-E. Other than the main incoming axons from labeled TG en route to deep layers, we did not observe any tomato-labeled Aβ axon collaterals or boutons sprouting into either the IB4 positive lamina II or the more superficial lamina I, where peptidergic nociceptive fibers terminate. This was observed in both the CFA-treated and the control side (Figure 1C-E, N = 5 mice). In samples collected 10 days after CFA injection (Figure 1F), we also failed to observe any ectopic sprouting of tomato-labeled axons into the noci-receptive layers of the SpC (Figure 1G-I, N = 5). Therefore, CFA-induced inflammatory pain did not cause abnormal central sprouting of Pv-expressing LTMR TG afferent axons into the superficial laminae.

### No evidence of ectopic central sprouting of TG Aβ touch afferents in a trigeminal neuropathic pain model

**Figure 2:**
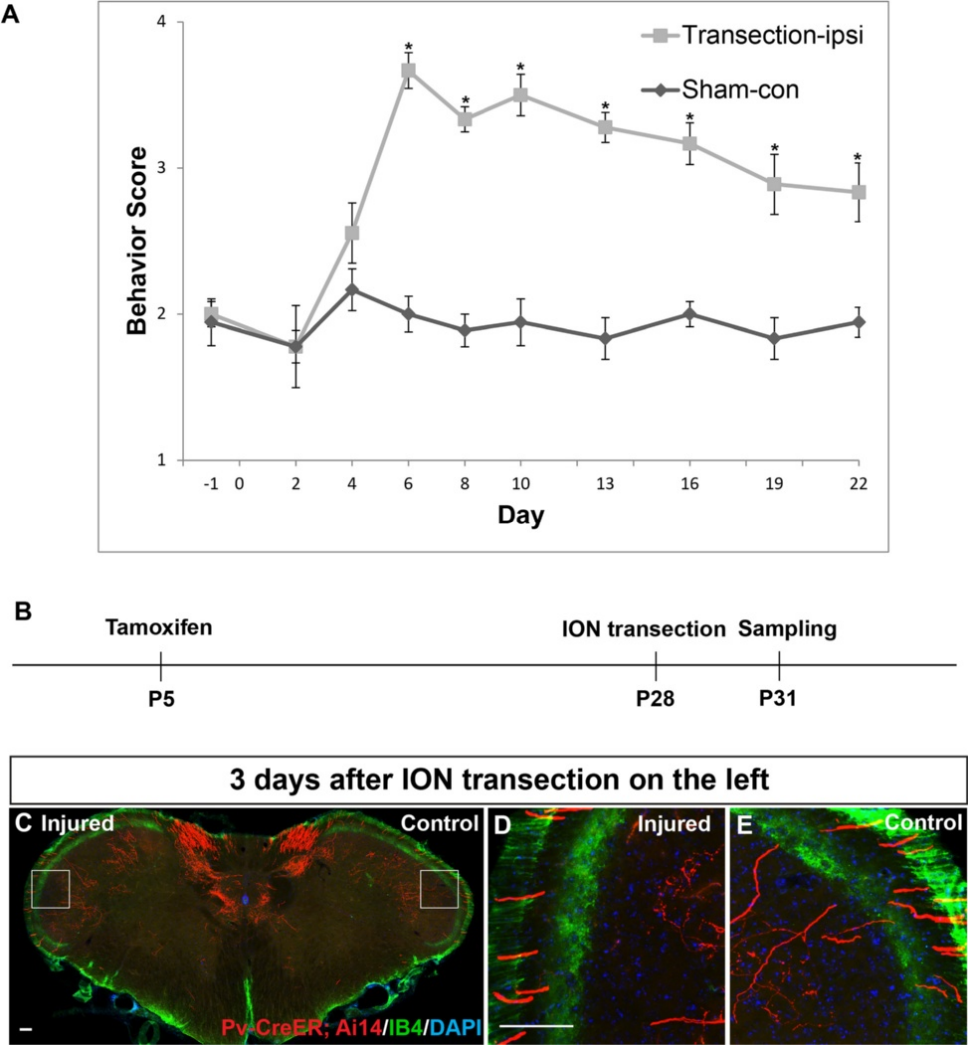
**ION transection caused long-lasting mechanical allodynia but no sprouting of labeled trigeminal Aβ afferents at the early phase of this neuropathic pain model. (A)** Unilateral ION transection induced mechanical allodynia in ipsilateral side lasting more than 3 weeks. Higher behavior score indicated an increased aversive response to von Frey filament stimulation. Results represent means ± SEM. P-values represent comparison to sham values (*P < 0.05). Differences were determined by Student’s *t* test between two groups, or one-way ANOVA followed by post-hoc Bonferroni test for multiple groups. n = 6 mice for each group. **(B)** Schematic of experimental procedure for results shown in **C-E**. **(C)** Representative spinal caudalis (SpC) section from Pv-CreER; Ai14 transgenic mice 3 days after ION injury on the left side, and sham operation on the right side. Red: labeled Aβ axon collaterals; Green: IB4-488 staining marked lamina II; Blue: DAPI. **(D-E)** High magnification of boxed areas in **C**. Scale bar: 100 μm.

**Figure 3:**
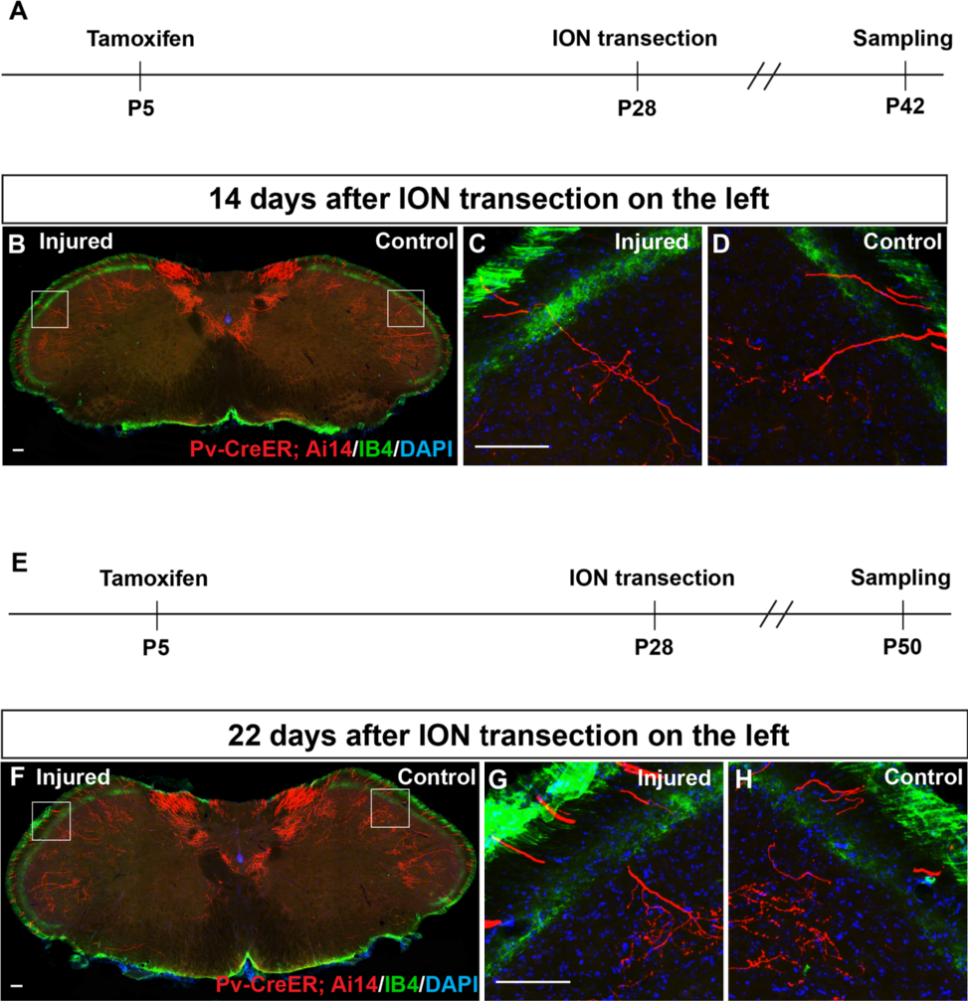
**Lack of ectopic sprouting from labeled trigeminal Aβ afferents at 14 and 22 days after ION transection. (A)** Schematic of experimental procedure for results shown in **B-D**. **(B)** Representative spinal caudalis (SpC) section from Pv-CreER; Ai14 transgenic mice 14 days after ION injury on the left side, and sham operation on the right side. Red: labeled Aβ axon collaterals; Green: IB4-488 staining marked lamina II; Blue: DAPI. **(C-D)** High magnification images of the boxed areas in B. **(E)** Schematic of experimental procedure for results shown in **F-H**. **(F)** Representative SpC section from Pv-CreER; Ai14 transgenic mice 22 days after ION injury on the left side. **(G-H)** High magnification images of boxed areas in F. Scale bar: 100 μm.

We also investigated the sprouting of Pv::CreER labeled LTMR TG afferents using a trigeminal neuropathic pain model, i.e. transection of the infraorbital nerve (ION) [4,9]. Although different studies reported different time course for the development of mechanical hypersensitivity after ION injury perhaps due to variations of the surgery and the extent of ligation/transection, we used method that produces a dramatic mechanical allodynia a few days after ION transection which lasts more than 3 weeks (Figure 2A) similar to that observed in other studies [37-39]. We chose to examine the axon arborizations at 3, 14, and 22 days after unilateral ION transection to cover both the early and the chronic phase of the neuropathic pain (Figures 2B and 3A and E, schematic of experimental procedure).

P5 “Pv::CreER; Ai14”-mice were injected with tamoxifen to specifically label individual TG Aβ afferents before injury. Subsequently, ION transection was performed on the left side, and sham operation was carried out on the right side in 4-week adult mice. 3, 14, 22 days after injury, mice were perfused and the SpC region of the samples were serial sectioned and stained with IB4-488. At all time points examined, we did not observe any labeled axon terminals sprouting into the IB4 positive or more superficial layers of the SpC (Figures 2 and 3, N = 5 mice for each group). High magnification images of the whisker representing regions in SpC are shown, and there were no differences observed between the control side and the injured side (Figures 2 and 3).

### Lack of peripheral sprouting of TG Aβ touch afferents in both CFA-induced inflammatory and neuropathic pain models

**Figure 4:**
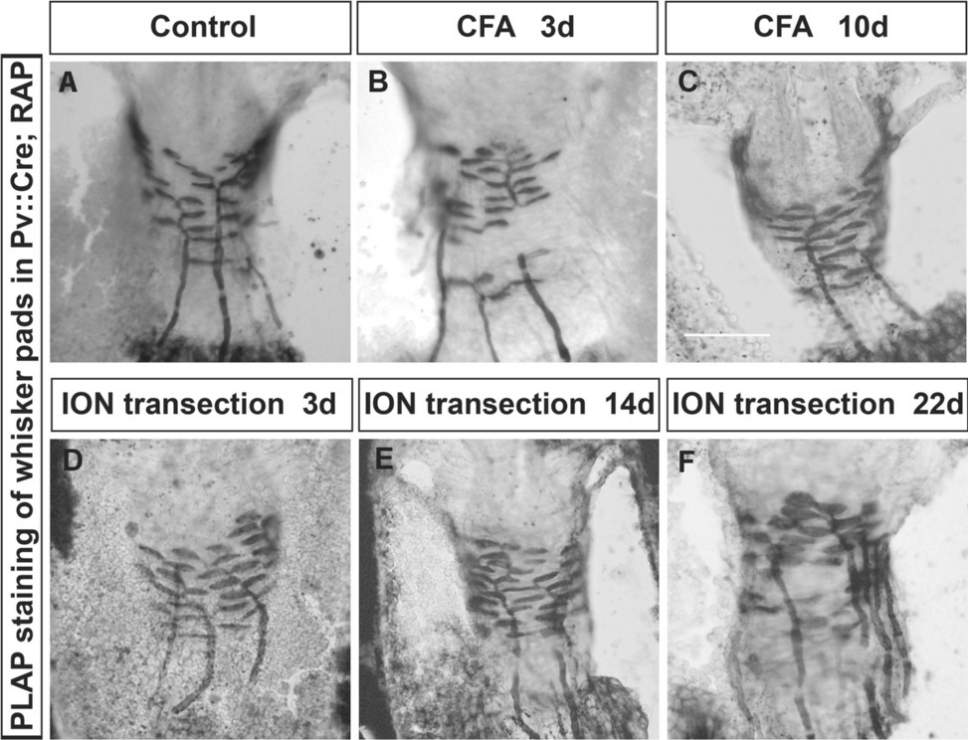
**Lack of peripheral axon terminals sprouting in whisker follicles in inflammatory or neuropathic pain models.** Representative images of AP stained whisker follicles from Pv::Cre; RΦAP mice showing labeled Aβ sensory endings. **(A)**: control condition; **(B-C)**: 3, and 10 days after CFA injection; **(D-F)**: 3, 14, and 22 days after ION injury. Scale bar: 100 μm.

With lack of evidence for central sprouting of Aβ axons, we next examined whether there might be any peripheral axonal sprouting of TG Aβ touch neurons. To do this, we crossed Pv::Cre with a PLAP reporter (RΦAP) mouse line which permitted efficient staining of peripheral axon terminals [26]. We examined the peripheral endings at day 3 and day 10 post-CFA injection, or at day 3, 14, 22 after ION transection. We observed disc-like Merkel endings and longitudinally extending axons inside the whisker follicles in both the injured and the control side. In all animals examined (N = 3 for each treatment at each time point), we did not observed any abnormal sprouting from the peripheral axons of Pv + neurons into superficial skin (Figure 4). Thus, the morphology of peripheral nerve endings was not changed in either inflammatory or neuropathic trigeminal pain models (Figure 4).

## Conclusions

Previous studies using intra-axonal labeling of DRG neurons, combined with electrophysiology did not support the hypothesis that Aβ afferents ectopically sprout into the superficial noci-receptive laminae of the spinal cord [11,13]. However, whether there was transient Aβ sprouting remains unknown because the analyses were performed at one time point in the later stages after nerve injury. There is also a possibility that some LTMR Aβ neurons undergo a complete phenoptypic change into HTMRs that project throughout lamina I-V after injury. These two important issues can be resolved by genetically pre-labeling of Aβ afferents and examining the central projections of labeled neurons after injury.

In this study, we selectively labeled a subset of Aβ low-threshold trigeminal touch neurons prior to inflammatory or neuropathic injuries, using a genetic labeling strategy relying on the Pv:CreER mouse line. Our genetic method allowed clear visualization of central axonal arborizations from the Aβ LTMR touch neurons. Subsequently, the labeled animals were examined at several time points either after inflammatory or nerve ligation-mediated injury. We found no evidence of ectopic peripheral or central axonal sprouting, either transiently or in more chronic conditions in all animals examined. Thus, the long lasting mechanical allodynia in our orofacial pain models is unlikely caused by ectopic Aβ axonal sprouting into “pain-processing” layers of the trigeminal spinal nucleus. Since we only labeled Pv-expressing Aβ TG neurons, we cannot rule out the possibility that other types of Aβ LTMR touch neurons that were not labeled in our study sprout axons after injury. However, our results together with previous studies strongly argue against the ectopic sprouting hypothesis. Instead, we favor the idea that gene expression changes in primary sensory neurons and/or sensitization or disinhibition of central processing circuits contribute more significantly to the tactile hypersensitivity [40,41].

## Materials and methods

### Mouse

Pv::CreER; Ai14 (Cre-dependent TdTomato reporter) and Pv-Cre; RΦAP adult male mice of at least 4-week old were used for injury models and subsequent axon projection studies. Both alleles are heterozygous. Pv::Cre mice (stock number 008069) and Ai14 (stock number 007914) were purchased from the Jackson Laboratories. RΦAP and Pv::CreER mice have been described previously [26,42]. All animal procedures were approved by The Duke University Institutional Animal Care and Use Committee.

### Tamoxifen injections

The tamoxifen solution dissolved in corn oil (0.05 mg/g; body weight) was injected subcutaneously in Pv-CreER; Ai14 pups at P5.

### Complete Freund’s Adjuvant (CFA) injection

All mice were briefly anesthetized with 2% isoflurane and injections were performed using a 30-G needle on a 10 μl Hamilton syringe. 10 μl of complete Freund adjuvant (CFA, 5 mg/ml; Chondrex, Redmond, WA) were injected into the left (ipsilateral) whisker pad. Control sides were injected with the same volume of incomplete Freund’s adjuvant (IFA, Chondrex). Following CFA treatment, the mice were subjected for behavior tests, and were sacrificed either at day 3 and 10 respectively for histology analysis.

### Infraorbital Nerve (ION) injury model

Mice were anesthetized with Ketamine/Xylazine (Sigma, 40 mg/3 mg/kg, i.p.), and all surgeries were performed in sterile conditions under a surgical microscope [4,9]. The infraorbital muscle was gently dissected from the bone until the orbit could be gently retracted. The infraorbital nerve on the left was dissected free from the bone at its most rostral extent in the orbital cavity. The nerve was then ligated with chromic gut suture (6–0, Angiotech) and transected at the point just distal to the ligature. For the sham operation on the right side, only skin incision and muscle dissection were performed, and the nerve was not touched and no chromic gut suture was inserted. All skin incisions were sutured with 5–0 nylon non-absorbable monofilament (Angiotech), and mice were subjected for behavior tests after surgery, and were sacrificed on day 3, 14 and 22 respectively for histology analysis.

### Behavioral testing

Mice after CFA injection or ION transection injury were subject to von Frey hair testing. Briefly, two different von Frey filaments (0.4 and 2.0 g) were used with the smallest hair used first. The response to each of the filaments is scored as follows: score 0, no response; score 1, detection - the mice detected and explored the von Frey hair; score 2, head slowly withdrawal reaction; score 3, escape/attack; score 4, asymmetric face grooming – at least three face wash strokes directed toward the stimulated facial area [37]. Three applications were performed for each filament and the mean of the measurements is used. All data were expressed as mean ± SEM. Differences between groups were evaluated using 2-tailed Student’s *t* test (experimental against sham control), or in the case of multiple groups, one-way ANOVA followed by post-hoc Bonferroni test. The criterion for statistical significance was p < 0.05.

### Histology

IB4-staing and placental alkaline phosphatase staining (PLAP staining) were performed using standard procedures. For PLAP staining, the tissue sections were incubated in PBS at 65°C for 3 hours, and developed with 1:200 NBT/BCIP (Roche) in staining solution which contains 0.1 M Tris–HCl, pH 9.5, 0.1 M NaCl and 5 mM MgCl_2_. GS*-*IB4 Alexa 488*-*conjugated (Invitrogen) was used at 1:1000.
